# Supplementary figures and images for: Distinctive courtship phenotype of the Vogelkop Superb Bird-of-Paradise Lophorina niedda Mayr, 1930 confirms new species status
Source: PeerJ. 2018 Apr 16;6:e4621. doi: 10.7717/peerj.4621 (PMC5907773; doi:10.7717/peerj.4621)

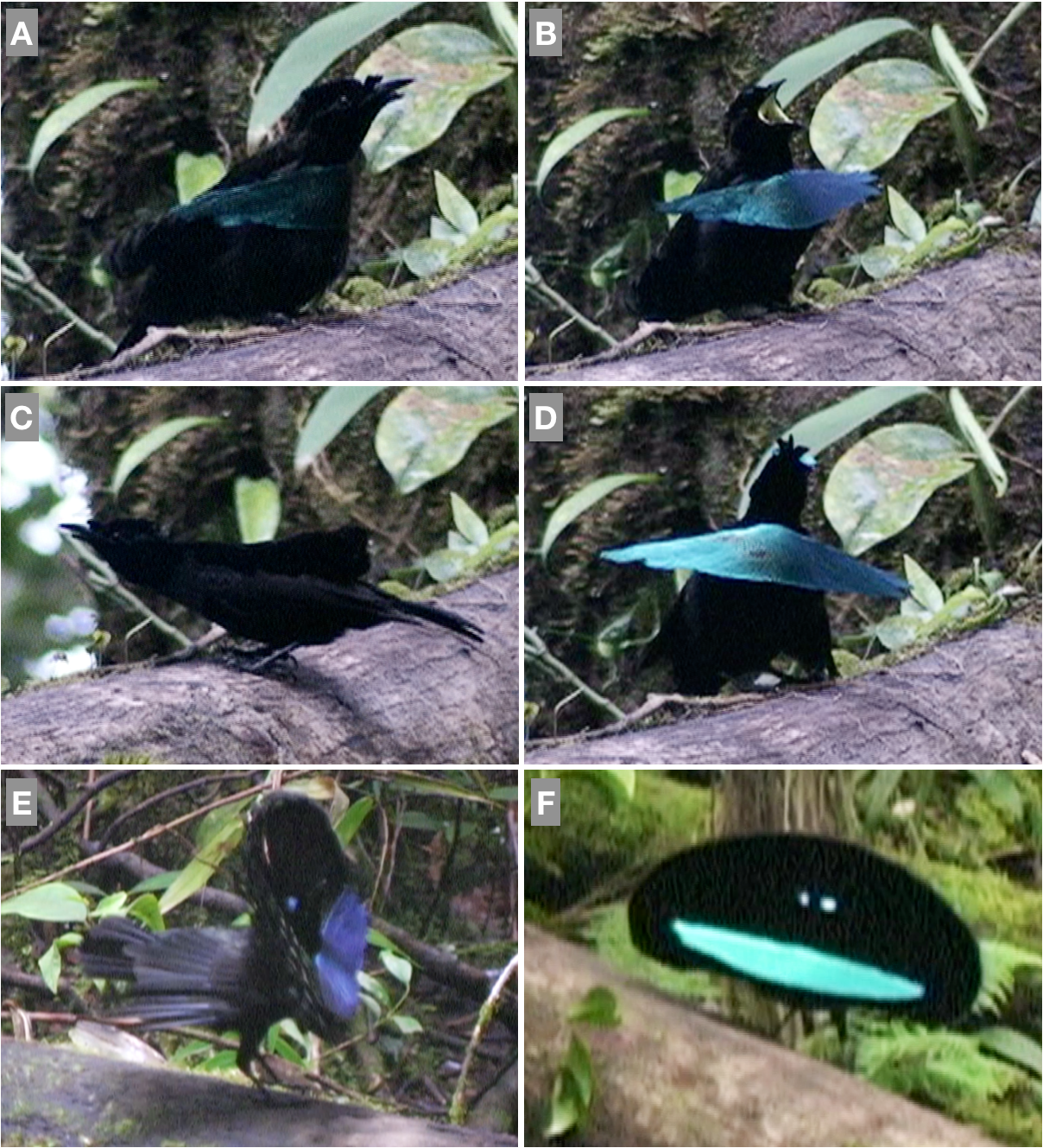

Supplement: Figure S1 — (A) Advertisement display and (B) vocalizing. (C) Horizontal display. (D) Pointing display. (E) Side view of the cape presentation display. (F) Frontal view of the cape presentation display. At the level of detail analyzed here, we found no discernible differences between the courtship phenotypes of superba superba and superba latipennis. Image credit/source: (A–D) Edwin Scholes/ML458167, (E) Kimberly Bostwick/ML487258 and (F) Kimberly Bostwick/ML487259. [file peerj-06-4621-s001.png]
